# Cefepime Alleviates Comorbid Pain and Depression Induced by Lipopolysaccharide in Female Mice

**DOI:** 10.3390/brainsci16030306

**Published:** 2026-03-12

**Authors:** Amna Khan, Patrick J. Ronan, Shafiqur Rahman

**Affiliations:** 1 Department of Pharmaceutical Sciences, College of Pharmacy, South Dakota State University, Brookings, SD 57007, USA; 2 Research Service, Sioux Falls VA Healthcare System, Sioux Falls, SD 57105, USA; 3 Department of Psychiatry and Basic Biomedical Sciences, University of South Dakota Sanford School of Medicine, Sioux Falls, SD 57105, USA

**Keywords:** glia, neuroinflammation, glutamate transporter, pain, depression, cefepime

## Abstract

**Background/Objectives**: Evidence indicates that aberrant glutamate transporter function and expression are linked to the pathophysiology of comorbid major depressive disorder (MDD) and pain. We have previously reported that cefepime (CFP) attenuates lipopolysaccharide (LPS)-evoked pain and depression by regulating hyperglutamatergic activity in male mice. However, the effects of CFP on LPS-evoked pain, depression-related anxiety, and cognitive impairment in female mice regarding sex-specific glial mechanisms remain unknown. **Methods**: Using behavioral paradigms, we evaluated the therapeutic potential of CFP in mitigating LPS-evoked pain, depression-related anxiety, and cognitive impairment in female mice. Furthermore, we used Western blot analysis to examine the effects of CFP on ionized calcium-binding adaptor molecule 1 (Iba-1) and glutamate transporter 1 (GLT-1) protein levels in the prefrontal cortex (PFC) and hippocampus (HPC). We also measured tumor necrosis factor-α (TNF-α) and interleukin-1β (IL-1β) concentrations in the HPC and PFC after CFP treatment using ELISA. **Results**: Pretreatment with CFP significantly increased the mechanical threshold and withdrawal latency in female mice. Additionally, systemic treatment with CFP markedly reduced immobility during the forced swim and tail suspension tests. Moreover, pretreatment with CFP remarkably augmented the open arm time during elevated plus maze test and spontaneous alternation between arms during Y-maze test. Western blot analysis indicated that systemic administration of CFP significantly reversed the downregulation of astroglial GLT-1 expression and reduced the microglial Iba-1 protein levels in the HPC and PFC. Furthermore, pretreatment with CFP significantly attenuated the LPS-evoked increase in the production of pro-inflammatory cytokines in the HPC and PFC. **Conclusions**: These results represent the novel inaugural report of a combined pain-MDD phenotype in female mice. The findings imply that positive glutamate transporter modulator CFP could be a novel treatment for comorbid pain and MDD in female patient population.

## 1. Introduction

The co-occurrence of pain and major depressive disorder (MDD) is a widespread neuropsychiatric disorder with a large socioeconomic burden, affecting over 300 million people worldwide [1]. Moreover, the coexistence of pain and depression mainly involves similar neurochemical, neuroglial, and morphological changes in the subcortical and cortical regions of the brain regulating cognitive and emotional aspects of pain [2,3]. Additionally, there is a well-recognized overall sex difference in the progress and symptomology of this neuropathological condition, with females outnumbering males [4]. It is worth mentioning that an abundance of preclinical evidence implicates the use of male mice only and the overlooking of sex differences, an important consideration when evaluating such neuropsychiatric symptoms. The lipopolysaccharide (LPS) model of nociceptive pain and depressive-like behaviors is a well-established animal model used to mimic not only behavioral features associated with nociceptive pain and depressive-like behaviors but also the effects of sex on these outcomes [5,6,7].

Compelling evidence demonstrates that inflammation confers hyperactive glutamatergic transmission at the neuronal synapse in the prefrontal cortex (PFC) and hippocampus (HPC) due to the activation of glial cells [8]. Moreover, inflammation mediated release of pro-inflammatory cytokines by activated microglia in the brain or from peripheral immune cells leads to increased concentrations of glutamate by impairing its removal via reuptake mechanisms and by promoting glutamate release [9]. This is partly accomplished through inflammatory mediators downregulating the expression and function of excitatory amino acid transporter 2 (EAAT-2) or rodent homologue glutamate transporter 1 (GLT-1) predominantly expressed on astrocytes [10]. Furthermore, postmortem studies have indicated decreased expression of numerous genes associated with synthesis of glutamate transporter in the brain of depressed subjects [11,12]. Importantly, decreased number and functioning of astroglial glutamate transporters have also been detected in postmortem studies of patients with depression [11,13,14]. In addition to excessive glutamatergic activity, potential effects of inflammatory mediators on microglia might be of relevance for the development of mood and cognitive abnormalities that overlap with those found in nociceptive pain [15,16]. Indeed, it should be noted that peripheral cytokine signals can activate microglial cells that amplify central inflammatory responses [17]. Interestingly, studies indicate that central inflammation drives microglial glutamate release by downregulating GLT-1, which inhibits astroglial glutamate uptake and triggers the production of pro-inflammatory cytokines [18]. Overall, these findings imply that inflammation-induced microglial activation and glutamate dysregulation are a major causal contributor to the onset of comorbid nociceptive pain and depression.

To date, five EAATs have been identified in humans and among these transporters, EAAT-2 or rodent homologue GLT-1 is predominantly expressed on astrocytes [19]. An emerging body of literature reveals that aberrant expression or malfunctioning of GLT-1 or EAAT-2 in humans can cause mood-related disorders [20]. Several pharmacological agents have been shown to modulate GLT-1 expression and have been found effective in treating various neuropsychiatric disorders. In this regard, an experimental drug, LDN-212320 (LDN) and beta-lactam antibiotics belonging to third generation cephalosporins including ceftriaxone (CFX) have been shown to enhance GLT-1 expression and subsequent glutamate reuptake, thereby exerting neuroprotective effects in neurological disorders [21]. It is important to state that LDN is a translational EAAT-2/GLT-1 activator that enhances glutamate uptake, restores synaptic integrity, and reduces glutamate-mediated excitotoxicity. Interestingly, cefepime (CFP), a fourth-generation cephalosporin has demonstrated neuroprotective effects across a wide range of preclinical models [22,23,24,25,26]. More recently, we reported that CFP attenuates LPS-evoked pain and depression by regulating hyperglutamatergic activity in male mice [27]. However, the effects of CFP on pain, depression-related anxiety, and cognitive impairment evoked by LPS in female mice, and the underlying sex-specific glial mechanisms have not been examined. Addressing a gap in the current scientific literature, this work provides the first report of a comorbid pain-MDD model in female mice.

In the current study, we have evaluated the effects of CFP on LPS-evoked mechanical hypersensitivity, thermal hyperalgesia, depression-related anxiety, and cognitive impairment in female mice using behavioral paradigms. Moreover, we have evaluated the effects of CFP on ionized calcium-binding adaptor molecule 1 (Iba-1) and GLT-1 protein levels in the HPC and PFC using Western blot. We also investigated the effects of CFP on tumor necrosis factor-α (TNF-α) and interleukin-1β (IL-1β) levels in the HPC and PFC using enzyme-linked immunosorbent assay (ELISA).

## 2. Materials and Methods

### 2.1. Animals

Sixty female C57BL/6J mice (7–8 weeks old, 20–30 g) were bought from Jackson laboratories (Bar Harbor, ME, USA). The mice were kept (four per cage) in a facility maintained at 22 ± 2 °C, 60% relative humidity, and a 12-h light/dark cycle (lights on at 0700 h) with food and water *ad libitum*. All behavioral studies were performed during the light cycle. Studies were conducted in accordance with Good Laboratory Practice and ARRIVE guidelines.

### 2.2. Drugs and Treatment

CFP hydrochloride, CFX sodium, and LPS (serotype 012:B8) were obtained from Sigma Aldrich (St. Louis, MA, USA). LDN was purchased from Axon MedChem (Reston, VA, USA). We have included LDN and CFX as positive controls for translational and transcriptional GLT-1 activation respectively, due to their analgesic [28,29,30], neuroprotective, and pro-cognitive properties [31,32]. All treatments were given intraperitoneally (i.p.) at 10 mL/kg bodyweight.

### 2.3. Experimental Design

**Figure 1:**
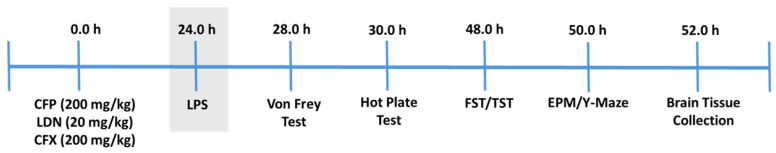
Study timeline. Mice received CFP (200 mg/kg), CFX (200 mg/kg), and LDN (20 mg/kg) 24 h prior to LPS injection. Behavioral tests were carried out to assess mechanical hypersensitivity and thermal hyperalgesia 4 h and 6 h post-LPS, respectively. In addition, behavioral tests were performed to determine depression, anxiety, and cognitive impairment 24 h and 26 h after LPS, respectively. The brain tissues were dissected for further analysis, 28 h post-LPS.

Animals were assigned to different experimental groups in a randomized way and administered LPS (1 mg/kg) to induce a systemic inflammatory response as described previously [33]. The mice in the treatment groups received LDN (20 mg/kg), CFX (200 mg/kg), and CFP (200 mg/kg) 24 h before LPS injection. Control animals were treated with an equal volume of saline (0.9% NaCl) in a similar manner. We selected a single dose of CFP (200 mg/kg) based on our previously reported data [27]. Behavioral tests to assess mechanical hypersensitivity and thermal hyperalgesia were carried out at 4 h and 6 h, post-LPS, respectively [34]. Behavioral tests to determine depression, anxiety, and cognitive impairment were performed 24 h and 26 h after LPS, respectively [35]. Three separate cohorts of animals were employed to investigate depression, anxiety, and cognitive impairment to avoid the confounding effects of handling stress on behavioral measures. In all the cohorts, each experimental group consisted of 4 animals. However, the von Frey, hot plate, forced swim test (FST), and elevated plus maze (EPM) tests were performed in the first two cohorts, whereas the tail suspension test (TST) and the Y-maze tests were conducted in the last cohort only (Figure 1). Animals were excluded from the study if they met the following criteria: excessive movement during the von Frey test, failure to respond within 30 s during the hot plate test, inability to maintain buoyancy in the FST, excessive tail-climbing behavior in the TST, or total immobility during the EPM and Y-maze tests. Behavioral videos were analyzed using ANY-maze software (v7.37, Stoelting Co., Wood Dale, IL, USA). Following behavioral experiments, mice were euthanized via decapitation. The brain tissues were promptly removed and kept at −80 °C for molecular and biochemical evaluation.

### 2.4. Behavioral Tests

#### 2.4.1. Von Frey Test

The von Frey test was performed [36], using a series of calibrated von Frey filaments and wire mesh boxes. Mechanical hypersensitivity was assessed 4 h after LPS injection and the tests were carried out on the mice in all groups. The paw withdrawal threshold was measured by applying each filament in a series of ascending forces to the sole of the hind paw 5 times for a period of 2 to 3 s. A positive response was considered if the withdrawal reflex was observed at least 3 times during the 5 applications.

#### 2.4.2. Hot Plate Test

The hot plate test was performed using an analgesia meter (IITC Life Sciences Inc., Woodland Hills, CA, USA) 6 h post LPS [37]. Briefly, each mouse was placed on a metal surface kept at 54.0 ± 0.1 °C with a maximum latency (cut-off) of 30 s. Hind paw withdrawal, licking, or shaking was interpreted as positive nocifensive behavior.

#### 2.4.3. Forced Swim Test

The FST was used to assess depressive-like behaviors 24 h after LPS administration and was performed in a Plexiglas container (20 cm diameter × 45 cm height) filled with 25 cm of water (25 ± 2 °C). Six-minute test sessions were conducted, and the immobility time was determined, as previously reported [38].

#### 2.4.4. Tail Suspension Test

For the evaluation of the effects of treatments on depressive-like behaviors, the animals were subjected to the TST as described previously [38] 24 h after LPS administration. Mice were individually suspended by their tails, in such a position that they cannot reach any nearby surfaces and their behavior was recorded to measure immobility time over a six-minute time period.

#### 2.4.5. Elevated Plus Maze Test

The EPM test was used to determine the effects of treatments on anxiety-like behaviors 26 h after LPS injection and was carried out in a four-armed maze (two open and two closed) elevated 40 cm above the floor. Five-minute test sessions were carried out, and the open arm time was determined, as described previously [28].

#### 2.4.6. Y-Maze Test

The Y-maze test was used to assess cognitive functions related to working memory 26 h after LPS administration. Animals were acclimated to the maze by being placed in the center and allowing free exploration of all arms. Five-minute test sessions were conducted, and the percentage of spontaneous alternations was determined, as described previously [38].

### 2.5. Western Blot Analysis

We conducted Western blot analysis as described previously [39]. Concisely, sixty micrograms of brain tissue samples were electrophoresed onto 12% SDS polyacrylamide gel using electrophoresis unit and transferred to a nitrocellulose membrane at 100 V for 1.5 h. Following blocking, membranes were incubated overnight at 4 °C with primary antibodies against GLT-1, Iba-1, and β-actin obtained from Santa Cruz Biotechnology, Dallas, TX, USA, followed by a 1 h incubation with HRP-conjugated secondary antibodies at room temperature (Table 1). After incubation, signals were visualized using ECL Select (Amersham, Buckinghamshire, UK), and the protein bands were quantified via densitometric analysis. β-actin was used as a loading control. Lane normalization factor was determined by dividing β-actin band intensity in each lane by the highest β-actin band intensity on the blot. Relative expression was calculated as the ratio of target protein band intensity by the lane normalization factor.

### 2.6. ELISA

The brain tissues were processed as reported previously [34]. Hippocampal and prefrontal cortical TNF-α and IL-1β were quantified via ELISA (Invitrogen Corporation, Waltham, MA, USA, Catalog No. 50-112-8954 and 50-112-9749), in accordance with the manufacturer’s instructions.

### 2.7. Statistical Analysis

Statistical significance was determined using one-way analysis of variance (ANOVA) followed by Tukey’s post hoc test for normally distributed data. A Kruskal–Wallis test followed by Dunn’s multiple comparison test was used to analyze behavioral data (von Frey and hot plate tests), which were not normally distributed. All statistical analyses were performed with GraphPad Prism (v10.0, GraphPad Inc., San Diego, CA, USA). Data were presented as the mean ± SEM. *p* < 0.05 was considered to be statistically significant.

## 3. Results

### 3.1. Effects of CFP on Nociceptive Behaviors in LPS-Injected Female Mice

**Figure 2:**
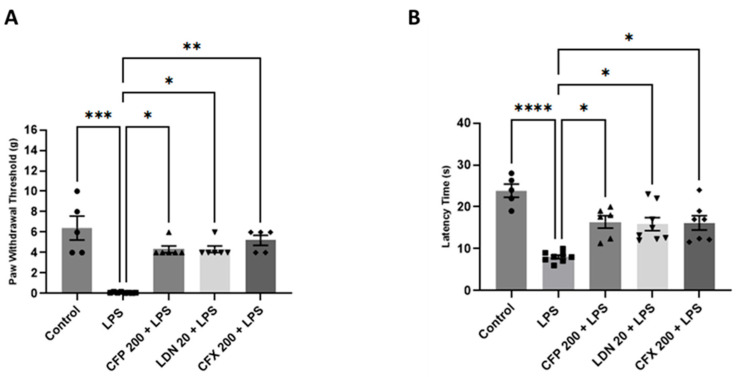
Effects of CFP on LPS-evoked nociceptive behaviors. (**A**) Effects of CFP (200 mg/kg) on mechanical threshold (g) during LPS-evoked mechanical hypersensitivity. (**B**) Effects of CFP (200 mg/kg) on withdrawal latency (s) during LPS-evoked thermal hypersensitivity. Data are presented as the mean ± SEM (*n* = 5–8); * *p* < 0.05, ** *p* < 0.01, *** *p* < 0.001, and **** *p* < 0.0001.

Compared to vehicle-injected controls, mice treated with LPS (1 mg/kg) exhibited marked nociceptive hypersensitivity, demonstrated by a significant reduction in mechanical threshold (*p* < 0.001; Figure 2A) and withdrawal latency (*p* < 0.0001; Figure 2B) during von Frey and hot plate tests. On the contrary, pretreatment with CFP (200 mg/kg) demonstrated an antinociceptive effect (*p* < 0.05; Figure 2A and *p* < 0.05; Figure 2B), reversing LPS-evoked mechanical hypersensitivity [H (4) = 21.35, *p* = 0.0003; Figure 2A] and thermal hyperalgesia [H (4) = 22.85, *p* = 0.0001; Figure 2B] compared to mice treated with LPS alone. Moreover, LDN pretreatment (20 mg/kg) significantly reversed pain hypersensitivity, as indicated by increased mechanical threshold (*p* < 0.05; Figure 2A) and withdrawal latency (*p* < 0.05; Figure 2B) relative to the LPS-injected mice. Furthermore, CFX (200 mg/kg) increased the mechanical threshold (*p* < 0.01; Figure 2A) and prolonged the withdrawal latency (*p* < 0.05; Figure 2B) relative to LPS control.

### 3.2. Effects of CFP on Depressive-like Behaviors in LPS-Injected Female Mice

**Figure 3:**
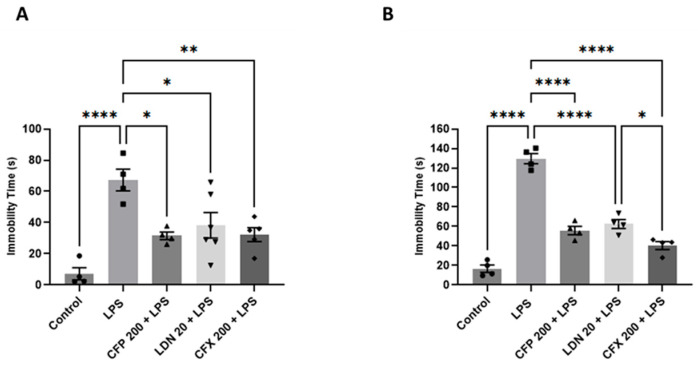
Effects of CFP on LPS-evoked depressive-like behaviors. (**A**) Effects of CFP (200 mg/kg) on immobility time (s) during FST. (**B**) Effects of CFP (200 mg/kg) on immobility time (s) during TST. Data are presented as the mean ± SEM (*n* = 4–6); * *p* < 0.05, ** *p* < 0.01, and **** *p* < 0.0001.

Intraperitoneal injection of LPS (1 mg/kg) evoked significant depressive-like behaviors in mice, evidenced by elevated immobility in the FST (*p* < 0.01; Figure 3A) and TST (*p* < 0.0001; Figure 3B) relative to controls. For the FST, CFP treated mice (200 mg/kg) showed significantly less immobility time (F_4,18_ = 10.29, *p* = 0.0002; Figure 3A) compared to the LPS group. Likewise, immobility time was significantly reduced (*p* < 0.05; Figure 3A) by LDN pretreatment (20 mg/kg) compared to the LPS-treated mice. Similarly, pretreatment with CFX (200 mg/kg) markedly reduced immobility compared to the LPS-injected mice (*p* < 0.01; Figure 3A). For the TST, there were significant effects of CFP (200 mg/kg), CFX (200 mg/kg), and LDN (20 mg/kg) on depressive-like behaviors evaluated as the time spent immobile (F_4,15_ = 89.46, *p* < 0.0001; Figure 3B).

### 3.3. Effects of CFP on Anxiety and Cognitive Impairment in LPS-Injected Female Mice

**Figure 4:**
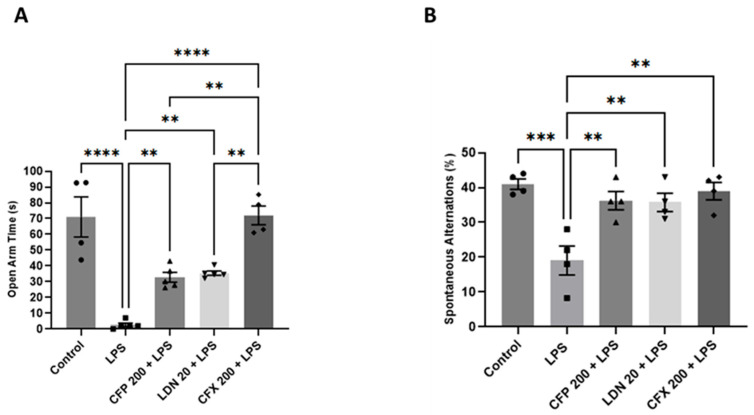
Effects of CFP on LPS-evoked anxiety and cognitive impairment. (**A**) Effects of CFP (200 mg/kg) on open arm time (s) during EPM test. (**B**) Effects of CFP (200 mg/kg) on spontaneous alternations (%) during Y-maze test. Data are presented as the mean ± SEM (*n* = 4–5); ** *p* < 0.01, *** *p* < 0.001, and **** *p* < 0.0001.

The LPS-treated group displayed reduced open arm time (*p* < 0.0001; Figure 4A) and spontaneous alternations (*p* < 0.001; Figure 4B) in the EPM and Y-maze tests, respectively relative to controls, demonstrating increased anxiogenic and cognitive deficit-like behaviors. However, CFP pretreatment (200 mg/kg) significantly reversed the reduction in open arm time (F_4,18_ = 26.19, *p* < 0.0001; Figure 4A) and spontaneous alternations (F_4,15_ = 9.662, *p* = 0.0005; Figure 4B) evoked by LPS. Thus, the group that received CFP (200 mg/kg) showed markedly augmented open arm time (*p* < 0.01; Figure 4A) and spontaneous alternations (*p* < 0.01; Figure 4B) in comparison to the LPS-treated mice. Moreover, LDN pretreatment (20 mg/kg) significantly elevated open arm time (*p* < 0.01; Figure 4A) and percentage of alternations (*p* < 0.01; Figure 4B), demonstrating the anxiolytic and procognitive effects. Furthermore, the CFX (200 mg/kg) treated mice showed a significant effect on open arm time (*p* < 0.0001; Figure 4A) and spontaneous alternations (*p* < 0.01; Figure 4B) relative to the LPS-injected mice.

### 3.4. Effects of CFP on GLT-1 Protein Levels in the HPC and PFC

**Figure 5:**
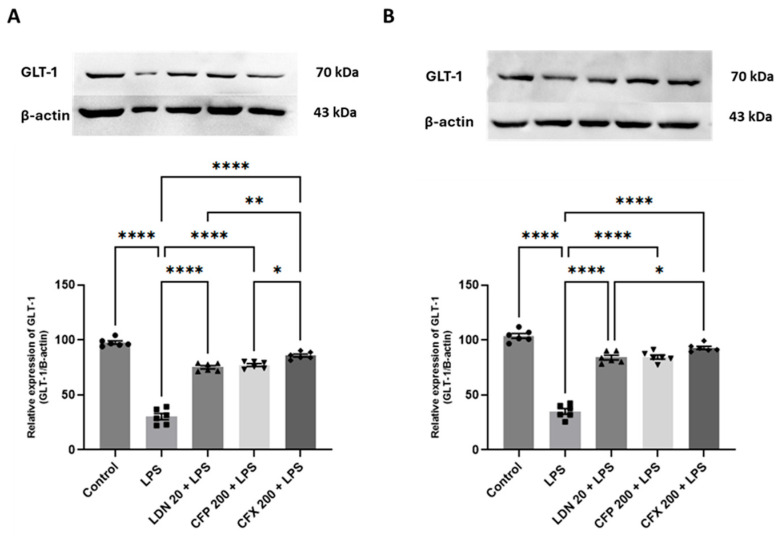
Effects of CFP on GLT-1 protein levels in the HPC and PFC. (**A**) Effects of CFP (200 mg/kg) on GLT-1 protein levels in the HPC. (**B**) Effects of CFP (200 mg/kg) on GLT-1 protein levels in the PFC. Data are presented as the mean ± SEM (*n* = 6); * *p* < 0.05, ** *p* < 0.01, and **** *p* < 0.0001.

Compared to vehicle-injected controls, LPS (1 mg/kg) noticeably reduced the GLT-1 protein levels in the HPC (*p* < 0.0001; Figure 5A) and PFC (*p* < 0.0001; Figure 5B). However, CFP pretreatment (200 mg/kg) reversed the LPS-decreased GLT-1 protein levels in the HPC (F_4,25_ = 201.8, *p* < 0.0001; Figure 5A) and PFC (F_4,25_ = 162.0, *p* < 0.0001; Figure 5B) relative to LPS control. Thus, the group that received CFP (200 mg/kg) showed markedly elevated GLT-1 protein levels in both the HPC (*p* < 0.0001; Figure 5A) and PFC (*p* < 0.0001; Figure 5B) relative to the LPS group. Moreover, LDN pretreatment (20 mg/kg) markedly modulated the LPS-evoked reduction in hippocampal (*p* < 0.0001; Figure 5A) and prefrontal cortical (*p* < 0.0001; Figure 5B) GLT-1 protein levels relative to LPS control. Furthermore, CFX pretreatment (200 mg/kg) remarkably augmented GLT-1 protein levels in the HPC (*p* < 0.0001; Figure 5A) and PFC (*p* < 0.0001; Figure 5B) compared to LPS control.

### 3.5. Effects of CFP on Iba-1 Protein Levels in the HPC and PFC

**Figure 6:**
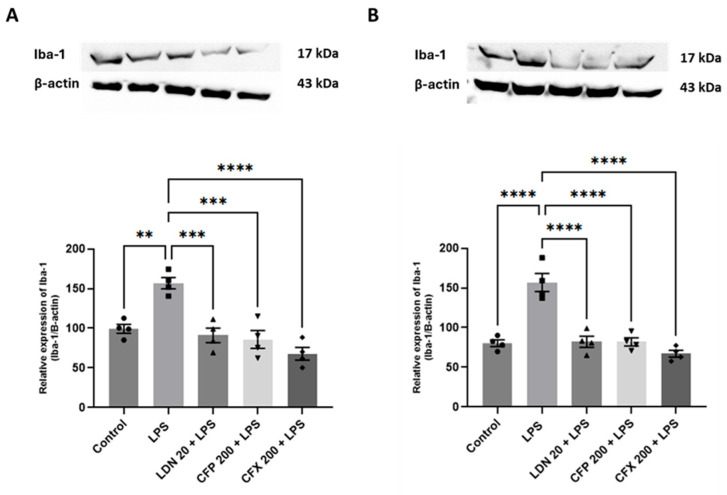
Effects of CFP on Iba-1 protein levels in the HPC and PFC. (**A**) Effects of CFP (200 mg/kg) on Iba-1 protein levels in the HPC. (**B**) Effects of CFP (200 mg/kg) on Iba-1 protein levels in the PFC. Data are presented as the mean ± SEM (*n* = 4); ** *p* < 0.01, *** *p* < 0.001, and **** *p* < 0.0001.

Compared to the control, LPS (1 mg/kg) noticeably augmented the Iba-1 protein levels in the HPC (*p* < 0.01; Figure 6A) and PFC (*p* < 0.0001; Figure 6B). Pretreatment with CFP (200 mg/kg) produced marked effects on Iba-1 protein levels in the HPC (F_4,15_ = 15.87, *p* < 0.0001; Figure 6A) and PFC (F_4,15_ = 26.73, *p* < 0.0001; Figure 6B) relative to LPS control. In addition, LDN (20 mg/kg) markedly attenuated the LPS-evoked elevation in Iba-1 protein levels in the HPC (*p* < 0.001; Figure 6A) and PFC (*p* < 0.0001; Figure 6B) relative to the LPS-treated group. Furthermore, pretreatment with CFX (200 mg/kg) remarkably abated Iba-1 protein levels in the HPC (*p* < 0.0001; Figure 6A) and PFC (*p* < 0.0001; Figure 6B) compared to LPS control.

### 3.6. Effects of CFP on TNF-α and IL-1β Production in the HPC and PFC

**Figure 7:**
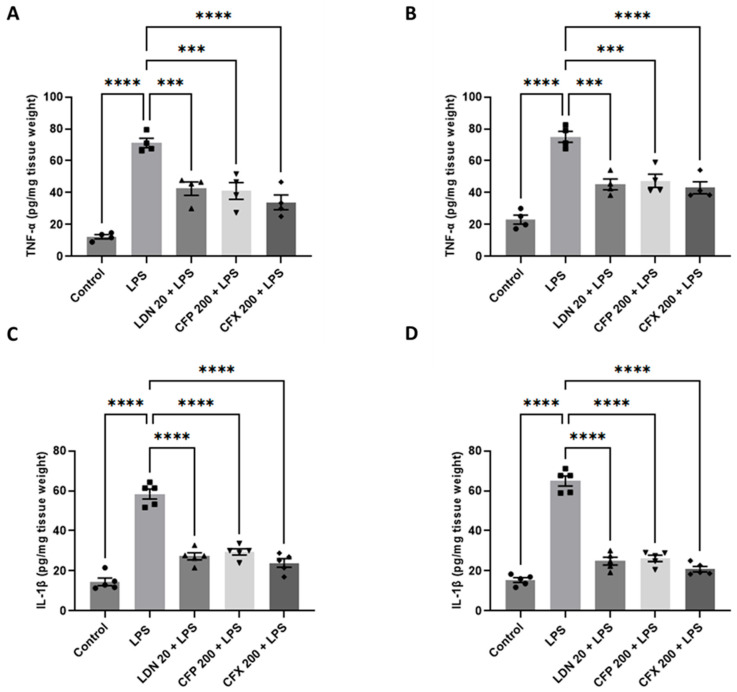
Effects of CFP on TNF-α and IL-1β production in the HPC and PFC. (**A**) Effects of CFP (200 mg/kg) on TNF-α production in the HPC. (**B**) Effects of CFP (200 mg/kg) on TNF-α production in the PFC. (**C**) Effects of CFP (200 mg/kg) on IL-1β production in the HPC. (**D**) Effects of CFP (200 mg/kg) on IL-1β production in the PFC. Data are presented as the mean ± SEM (*n* = 4–5); *** *p* < 0.001, and **** *p* < 0.0001.

Compared to the control, LPS (1 mg/kg) markedly increased the levels of TNF-α in the HPC (*p* < 0.0001; Figure 7A) and PFC (*p* < 0.0001; Figure 7B). However, CFP (200 mg/kg) remarkably attenuated the LPS-evoked increase in TNF-α production in the HPC (F_4,15_ = 28.74, *p* < 0.0001; Figure 7A) and PFC (F_4,15_ = 27.69, *p* < 0.0001; Figure 7B) compared to LPS control. Similarly, LDN (20 mg/kg) administration abated the LPS-evoked overproduction of TNF-α in the HPC (*p* < 0.001; Figure 7A) and PFC (*p* < 0.001; Figure 7B) compared to the LPS-treated group. In addition, CFX (200 mg/kg) also reduced the levels of TNF-α in the HPC (*p* < 0.0001; Figure 7A) and PFC (*p* < 0.0001; Figure 7B) in comparison to LPS control. Furthermore, intraperitoneal injection of LPS (1 mg/kg) noticeably elevated the levels of IL-1β in the HPC (*p* < 0.0001; Figure 7C) and PFC (*p* < 0.0001; Figure 7D) relative to the control. However, CFP (200 mg/kg) markedly reduced the production of IL-1β in the HPC (F_4,20_ = 69.18, *p* < 0.0001; Figure 7C) and PFC (F_4,20_ = 126.9, *p* < 0.0001; Figure 7D) compared to LPS control. Likewise, LDN (20 mg/kg) administration prevented the LPS-evoked overproduction of IL-1β in the HPC (*p* < 0.0001; Figure 7C) and PFC (*p* < 0.0001; Figure 7D) compared to the LPS-treated group. Furthermore, CFX (200 mg/kg) also reduced the production of IL-1β in the HPC (*p* < 0.0001; Figure 7C) and PFC (*p* < 0.0001; Figure 7D) in comparison to the LPS group.

## 4. Discussion

We observed that systemic treatment with CFP effectively mitigated mechanical hypersensitivity, thermal hyperalgesia, depression-related anxiety, and cognitive impairment in LPS-injected female mice. We have also found that systemic administration of CFP significantly modulated the glutamate transporter protein levels in LPS-treated female mice as revealed by increased GLT-1 expression in the HPC and PFC. Furthermore, intraperitoneal administration of CFP remarkably reversed the LPS-evoked elevation of Iba-1 expression, indicating the attenuation of microglial reactivity and subsequent release of pro-inflammatory cytokines in the HPC and PFC, brain regions regulating cognitive and emotional aspects of pain. The observed decrease in hippocampal and prefrontal cortical IL-1β and TNF-α was likely driven by reduced microglial activation, consistent with evidence that LPS induces robust microglial responses [40]. Increasing studies report that pro-inflammatory cytokine signaling in the brain, evoked by systemic LPS, causes pain sensitization and depressive-like behaviors in rodents [33,41]. Furthermore, pro-inflammatory cytokines have been shown to decrease astroglial glutamate transporter expression, causing reduced glutamate reuptake [42,43]. Our data suggest that LPS-evoked neuroinflammation, which is marked by elevated IL-1β and TNF-α, led to reduced GLT-1 protein levels in the HPC and PFC. To the best of our knowledge, this study provides the first evidence in a comorbid pain-MDD model in female mice.

Our findings demonstrate that intraperitoneal administration of LPS triggers neuroinflammation in limbic brain regions, which contributes to the development of nociceptive pain, depression-related anxiety, and cognitive impairment in female mice. Considerable evidence supports the notion that several brain regions including the HPC and PFC are involved in anxiety- and depression-related phenotypes in rodents and humans [44]. In addition, depression, anxiety, and cognitive abnormalities in painful conditions are acknowledged as being pain-related disabilities [45]. Furthermore, it is known that inflammatory signals are associated with changes in brain signaling patterns, which elicit depression, anxiety, and cognitive deficit-like behaviors [46].

We have found that intraperitoneal LPS injection causes the microglial activation and subsequent release of inflammatory mediators in the hippocampal and prefrontal cortical regions of female mice, which are prevented by systemic treatment with CFP. Previous studies have reported that LPS injection in humans can cause an increase in systemic pro-inflammatory cytokines and produces nociceptive hypersensitivity and depressive-like behaviors as a consequence of immune to brain communication [47], and that has been extensively studied in male rodent models [48,49,50]. Additionally, we have previously reported that CFP attenuates pain, depression-related anxiety, and cognitive impairment evoked by LPS via regulating microglial reactivity and hyperglutamatergic neurotransmission in male mice [27]. In this study, we found that systemic LPS administration causes mechanical hypersensitivity, thermal hyperalgesia, depression-related anxiety, and cognitive impairment in female mice associated with increased microglial activation and cytokine production in the HPC and PFC. In accordance with previous studies, these findings demonstrate that 24 h after a single LPS injection, microglial activation occurs, followed by an increase in the production of pro-inflammatory cytokines in the limbic brain regions [51,52]. Recent studies have revealed that LPS upregulates inflammatory cytokine expression and causes depressive-like behaviors in both male and female rodent models [53,54]. Prior research has also documented that dysregulated microglial activation profoundly contributes to the pathogenesis of comorbid pain and depression [55,56]. However, we cannot exclude the possibility that inflammatory mediators also compromise vital astrocytic homeostatic functions [57,58]. While our present study does not investigate whether astrocytes are activated and produce pro-inflammatory cytokines and exacerbate neuroinflammation in the HPC and PFC, prior findings strongly indicate that astrogliosis is a downstream effect of microglial activation [59,60]. Since microglia are highly susceptible to LPS exposure owing to their broad range of toll-like receptors (TLRs), astrocytes express these receptors minimally, resulting in low reactivity to LPS [61,62]. This underscores the vital role of microglia in triggering astrocytic responses.

Our results indicate that pretreatment with CFP markedly reversed the LPS-decreased GLT-1 protein levels in the HPC and PFC of female mice. Consistent with our findings, neuroinflammation has been shown to trigger microglial glutamate release via the downregulation of astroglial GLT-1 and subsequent suppression of glutamate uptake, leading to increased cytokine release [63,64]. Interestingly, evidence suggests that excessive glutamatergic neurotransmission associated with reduced functioning of GLT-1 participates in the pathogenesis of nociceptive pain and depressive-like behaviors [65]. Mounting evidence indicates that dysregulated glial function contributes to neuroanatomical and neurochemical abnormalities within the HPC and PFC, brain areas involved in pain modulation and emotion regulation [66,67]. It is important to state that EAAT-2/GLT-1 serves as the primary regulator of excitatory neurotransmission by maintaining low extracellular glutamate concentrations [68]. Furthermore, Hu et al. reported that upregulation of GLT-1 via systemic CFX treatment prevents hyperalgesia in an inflammatory mouse model [69]. Likewise, a recent study demonstrated that CFX alleviates anxiety- and depression-like behaviors in rats during ethanol withdrawal by regulating GLT-1 expression [32]. Previously, one study found higher expression of EAATs genes in the dorsolateral PFC of female subjects with depression but not in males [70]. Moreover, no upregulation of GLT-1 expression was observed in the cerebellum of LDN-injected females compared to males during stroke-associated brain injury [71]. Accordingly, the high degree of variability in disease model severity explains the sex-specific modulation of GLT-1 in distinct brain regions. Additionally, our findings demonstrate that LPS-evoked downregulation of hippocampal and prefrontal cortical GLT-1 is linked to increased production of inflammatory cytokines, suggesting that this glutamate transporter dysregulation drives pain, mood disorders, and cognitive deficits.

We have previously demonstrated that CFP attenuates LPS-evoked pain, depression-related anxiety, and cognitive impairment in a dose-dependent manner in male mice [27]. Accordingly, we have used CFP (200 mg/kg) for behavioral and biochemical/molecular analysis in the present study. Notably, we have documented that CFP is safe and efficacious at therapeutic doses and have already discussed recent findings concerning CFP linked to its neuroprotective effects [27]. In addition to that, we previously assessed the effects of CFP alone on LPS-evoked pain, depression-related anxiety, and cognitive impairment [27]. We also reported that dihydrokainic acid, a GLT-1 inhibitor abolished the neuroprotective effects of CFP, suggesting a critical role of the astroglial GLT-1 in producing these effects [27]. Likewise, our prior research indicates that systemic administration of CFP (200 mg/kg) alone augments GLT-1 expression at the transcriptional level in the HPC and PFC [27].

Substantial evidence demonstrates that glutamate transporter expression is dysregulated at both the transcriptional and translational levels in the HPC and PFC across various pain and depression animal models [72]. In this regard, glutamate transporters can be pharmacologically modulated by increasing their mRNA expression (transcription) and/or by increasing their protein levels (translation) [73]. The diverse regulatory mechanisms of GLT-1 offer multiple therapeutic avenues for neuroinflammatory conditions involving enhanced glutamatergic neurotransmission [72,73]. Accordingly, several GLT-1 activators, including the transcriptional agent CFX and the translational agent LDN, have been shown to provide neuroprotection across a range of neurological and psychiatric conditions [30,31]. It should be noted that transcriptional activators induce a sustained increase in GLT-1 mRNA, resulting in prolonged upregulation of GLT-1 expression [74]. In contrast, repeated administration of GLT-1 translational activators is frequently required to achieve a prolonged increase in glutamate transporter expression [21]. In this context, CFP induces transcriptional upregulation of GLT-1, thus providing a sustained mechanism to increase long-term glutamate transporter expression and function.

There are three major limitations in this study that could be addressed in future research. First, the study examined the effects of CFP on an acute inflammatory model. Second, although our data demonstrate significant astroglial GLT-1 modulation, the specific cellular, molecular, and region-specific mechanisms mediating this process, particularly in the context of pain, depression-related anxiety, and cognitive impairment are not yet fully understood. Third, instead of testing for a sex-treatment interaction, this study analyzed the sexes independently. Future research should investigate the effects of CFP on LPS-evoked chronic pain, depression-related anxiety, and cognitive impairment, with a focus on sex differences and other brain regions. Additionally, to ensure accurate reporting of sex differences, future studies should utilize mixed-sex cohorts and test for an interaction between sex and treatment [75].

## 5. Conclusions

**Figure 8:**
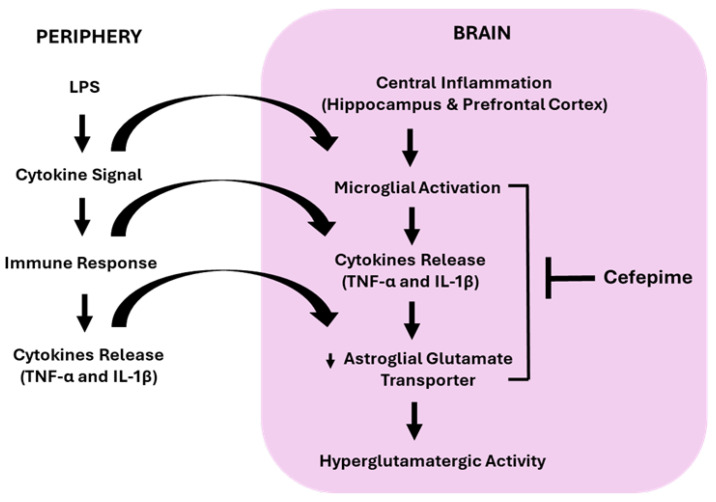
This figure illustrates the proposed mechanism of action of CFP on LPS-evoked pain, depression-related anxiety, and cognitive impairment. LPS induces the release of inflammatory mediators in the HPC and PFC. When activated, these inflammatory cytokines cause increased glutamatergic neurotransmission via downregulation of glutamate transporter 1. This hyperglutamatergic activity can be attenuated by administering positive glutamate transporter modulator CFP.

Our results provide compelling evidence that positive glutamate transporter modulator CFP alleviates LPS-induced mechanical hypersensitivity, thermal hyperalgesia, depression-related anxiety, and cognitive impairment in female mice. These effects are likely mediated by the modulation of microglial reactivity and hyperglutamatergic neurotransmission in the HPC and PFC (Figure 8). Furthermore, systemic treatment with CFP decreased the concentration of pro-inflammatory cytokines in the HPC and PFC by targeting glial mechanisms and the brain glutamatergic system. This study offers a novel approach by establishing a comorbid pain-MDD model in female mice. Thus, positive glutamate transporter modulator CFP could be a novel treatment for comorbid pain and MDD in female patient population.

## Figures and Tables

**Table 1:** List of names and catalog numbers of all antibodies used in Western blot.

Antibody	Dilution	Source	Catalog No.
GLT-1	1:1000	Santa Cruz	sc-365634
Iba-1	1:500	Santa Cruz	sc-32725
β-actin	1:1000	Santa Cruz	sc-47778
m-IgGκ BP-HRP	1:5000	Santa Cruz	sc-516102
m-IgG Fc BP-HRP	1:5000	Santa Cruz	sc-525409

## Data Availability

All data included in this study are available upon request from the corresponding author.

## Abbreviations

The following abbreviations are used in this manuscript:

CFP	Cefepime
CFX	Ceftriaxone
EAAT-2	Excitatory amino acid transporter 2
ELISA	Enzyme-linked immunosorbent assay
EPM	Elevated plus maze
FST	Forced swim test
GLT-1	Glutamate transporter 1
HPC	Hippocampus
Iba-1	Ionized calcium-binding adaptor molecule 1
IL-1β	Interleukin-1β
LDN	LDN-212320
LPS	Lipopolysaccharide
PFC	Prefrontal cortex
TLRs	Toll-like receptors
TNF-α	Tumor necrosis factor-α
TST	Tail suspension test
